# Global gene profiling of aging lungs in Atp8b1 mutant mice

**DOI:** 10.18632/aging.101056

**Published:** 2016-09-29

**Authors:** Ramani Soundararajan, Timothy M. Stearns, Alexander Czachor, Jutaro Fukumoto, Christina Turn, Emma Westermann-Clark, Mason Breitzig, Lee Tan, Richard F. Lockey, Benjamin L. King, Narasaiah Kolliputi

**Affiliations:** ^1^ Division of Allergy and Immunology, Department of Internal Medicine, Morsani College of Medicine, University of South Florida, Tampa, FL 33612, USA; ^2^ MDI Biological Laboratory, Salisbury Cove, ME 04672, USA; ^3^ University of Florida College of Medicine, Gainesville, FL 32608, USA; ^4^ Division of Allergy and Immunology, Department of Internal Medicine, James A Haley Veterans Hospital, Tampa, FL 33612, USA

**Keywords:** gene profiling, lungs, aging, transcriptome, Atp8b1 mutant

## Abstract

**Objective:**

Recent studies implicate cardiolipin oxidation in several age-related diseases. *Atp8b1* encoding Type 4 P-type ATPases is a cardiolipin transporter. Mutation in *Atp8b1* gene or inflammation of the lungs impairs the capacity of Atp8b1 to clear cardiolipin from lung fluid. However, the link between *Atp8b1* mutation and age-related gene alteration is unknown. Therefore, we investigated how *Atp8b1* mutation alters age-related genes.

**Methods:**

We performed Affymetrix gene profiling of lungs isolated from young (7-9 wks, n=6) and aged (14 months, 14 M, n=6) C57BL/6 and *Atp8b1* mutant mice. In addition, Ingenuity Pathway Analysis (IPA) was performed. Differentially expressed genes were validated by quantitative real-time PCR (qRT-PCR).

**Results:**

Global transcriptome analysis revealed 532 differentially expressed genes in *Atp8b1* lungs, 157 differentially expressed genes in C57BL/6 lungs, and 37 overlapping genes. IPA of age-related genes in *Atp8b1* lungs showed enrichment of Xenobiotic metabolism and Nrf2-mediated signaling pathways. The increase in *Adamts2* and *Mmp13* transcripts in aged *Atp8b1* lungs was validated by qRT-PCR. Similarly, the decrease in *Col1a1* and increase in *Cxcr6* transcripts was confirmed in both *Atp8b1* mutant and C57BL/6 lungs.

**Conclusion:**

Based on transcriptome profiling, our study indicates that *Atp8b1* mutant mice may be susceptible to age-related lung diseases.

## INTRODUCTION

Aging is associated with an overall decline in lung function, changes in lung physiology, and an increased susceptibility to diseases [1]. Lung injury and inflammation are associated with the aging process and are also observed during acute lung injury (ALI) [2]. As aging has been linked to the development of several lung diseases, understanding the underlying molecular and cellular mechanisms of aging is a pre-requisite for developing novel therapeutics for age-related lung diseases including, but not limited to chronic obstructive pulmonary disease (COPD), idiopathic pulmonary fibrosis (IPF), lung fibrosis, and lung cancer [2]. The process of normal physiological aging leads to enlarged alveolar spaces and loss of lung elasticity [3]. A combination of both intrinsic factors, such as age-dependent gene changes, and extrinsic factors, such as epigenetics and environmental stress, increase susceptibility to lung diseases [4]. Some of the age-associated features include increased oxidative stress, commonly in the form of reactive oxygen species (ROS, accumulation), alteration in the extracellular matrix (ECM), decreased production of anti-aging molecules, reduced antioxidant response, autophagy, and defective mitochondria [1, 4-6].

Mitochondria are the cellular power supply, providing energy required for cell survival. At the same time, mitochondria are involved in sensing danger signals and frequently cause inflammation by regulating the innate immune system [7]. Mitochondrial ROS have been implicated in the aging process and oxidative stress-induced pathology [8]. During normal physiological conditions, low levels of mitochondria-produced ROS serve as a redox sensor which regulates intracellular sig-naling and leads to cellular homeostasis [9], but excessive ROS can cause irreversible damage and result in mitochondrial dysfunction and eventually apoptosis [10].

Mitochondrial membrane phospholipids play an important role in the aging process as a result of modulating oxidative stress and molecular integrity [11]. Cardiolipin is a phospholipid that is localized to the inner mitochondrial membrane and is involved in regulating several mitochondrial bioenergetic processes, mitochondrial stability, and dynamics [11]. In lungs, the type II alveolar epithelial cells (AEC) secrete cardiolipin, a minor constituent of the alveolar surfactant, into airways [12]. In many pathological conditions, there is alteration in the structure and content of cardiolipin leading to mitochondrial dysfunction [13]. An example of this occurs during lung inflammation and injury, where an increased amount of cardiolipin is seen in the airways. This suggests that under normal conditions, the cardiolipin availability in extracellular fluid or airways is tightly regulated. Recent studies suggest that cardiolipin oxidation or age-related depletion leads to mitochondrial bioenergetic alteration, mitochondrial dysfunction, and eventually cell death [11, 13, 14].

Atp8b1 is a cardiolipin transporter and is critical for maintaining optimal cardiolipin levels in airways and extracellular fluids [15]. During inflammation, or when Atp8b1 is defective, its capacity to remove cardiolipin from lung fluid is impaired [15]. Mutations in the *Atp8b1* gene are associated with progressive familial intrahepatic cholestasis type 1 (PFIC1, or Byler's disease) [16]. These individuals are susceptible to pneumonia and respiratory symptoms [15]. *Atp8b1* mutant mice and humans with pneumonia have elevated levels of cardiolipin in lung fluid, which disrupts the function of the surfactant. Furthermore, cardiolipin dysregulation has been associated with aging [11]. However, the link between the *Atp8b1* mutation and age-related genes has not yet been studied. Therefore, the main aim of this study was to investigate how *Atp8b1* mutation alters age-related genes.

Global gene profiling in lungs is critical to developing a better understanding of the molecular and cellular events that are associated with aging of lungs. Transcriptome analysis in various mouse tissues has explored age-associated changes in gene expression and effects of caloric restriction [17, 18]. Few studies have determined the age-related changes in lung transcript-tome using mouse models [9, 10]. Therefore, in the present study, we used C57BL/6 mice and *Atp8b1* mutant mice (on C57BL/6 background) to study and compare age-dependent changes in gene expression.

The Affymetrix microarray analysis of lungs isolated from C57BL/7 mice at 7-9 wks and 14 M showed 157 differentially expressed genes associated with aging. Interestingly, 532 differentially expressed genes were linked to aging in *Atp8b1* mutant mice We identified 37 overlapping genes in two data sets, 85 unique genes in C57BL/7 lungs and 350 unique genes in *Atp8b1* mutant lungs. We validated several genes by qRT-PCR in *Atp8b1* mutant lungs including *Col1a1*, *Mmp13*, *Adamts2* and *CxCr6*. Similarly the transcripts *Col1a1* and *CxCr6* were also validated in C57BL/6 by qRT-PCR. Further, our gene profiling study indicates that distinct gene pathways, including xenobiotic metabolism and Nrf2 signaling pathway, are altered in *Atp8b1* mutant mice relative to C57BL/6 mice in an age-dependent manner. Taken together, these results suggest that *Atp8b1* mutant mice may be susceptible to age-related lung fibrosis, a phenotype associated with gene mutation and environmental trigger(s).

## RESULTS

### Age-related phenotype in *Atp8b1* mutant mice

Lungs are the primary organs where gas exchange takes place. On account of a high oxygen environment, the post-mitotic cells in lungs are susceptible to oxidative stress-related injury in an age-dependent manner [3]. This phenotype is shared among other tissues with high oxygen consumption rates including skeletal muscle, as well as heart and brain tissues [17, 18]. *Atp8b1* encodes a membrane-bound transporter Atp8b1 (ATPase, aminophospholipid transporter, class I, type 8B, member 1) that regulates AEC function. *Atp8b1* mutant mice carrying the mutation in the membrane-bound transporter showed decreased lung function and had an increased susceptibility to bacterial-induced pneumonia and epithelial cell apoptosis [15]. Therefore, we investigated the transcriptome of *Atp8b1* mutant lungs to study age-related changes in gene transcripts.

### Gene profiling studies revealed age-related genes in *Atp8b1* mutant and C57BL/6 lungs

**Figure 1:**
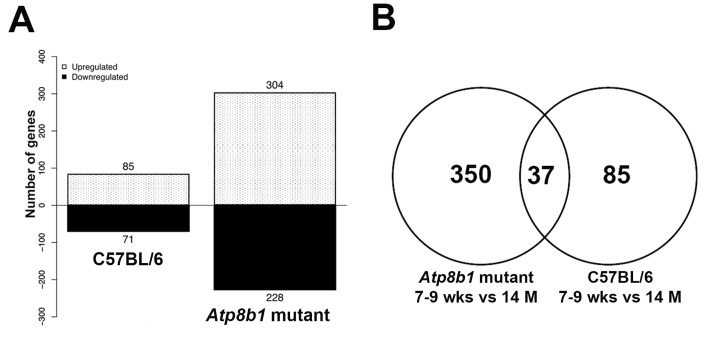
Differentially expressed genes in C57BL/6 and *Atp8b1* mutant lungs (**A**) Differentially expressed genes in the young (7-9 wk) (N=6) vs. aged (14 M) (N=6) C57BL/6 and *Atp8b1* mutant lungs from a total of 34,000 genes that were analyzed by Affymetrix microarray, respectively. p < 0.05. (**B**) Venn diagram depicting overlapping and unique genes in aged C57BL/6 and *Atp8b1* mutant lungs. Comparison of the differentially expressed genes (7-9 wks vs 14 M) in C57BL/6 and *Atp8b1* mutant lungs revealed 37 overlapping genes between the two datasets, 350 unique genes in *Atp8b1* mutant lungs and 85 unique genes in C57BL/6 lungs.

First, we performed microarray analysis in C57BL/6 lungs at 7-9 wks (N=6, adult) and 14 M (N=6, old) using Affymetrix Mouse 430v2.0 microarray. Gene profiling revealed 157 genes that were differentially expressed in an age-dependent manner. Of these, 85 genes were up-regulated and 71 genes were down-regulated in C57BL/6 lungs (Figure 1A). Some of the transcripts that were either increased or decreased with aging in C57BL/6 lungs included *Akap13*, *Calml3*, *Cdc73*, *Fbox15*, *Jak1, Midn*, *Mkx*, *Wif1, Wdpcp,* and *Zbtb16* (Table 1).

**Table 1:** Differentially expressed genes associated with aging in C57BL/6 lungs (q<0.1, mean-value difference between two groups >1.0)

Transcripts that are downregulated in C57BL/6 mice with aging						
Affymetrix Probe Set ID		Gene Symbol	Gene Name		Fold Change	FDR (q value)
1438239_at		Midn	midnolin		−3.737	0.0666
1439163_at		Zbtb16	zinc finger and BTB domain containing 16		−2	0.0903
1458423_at		Luc7l2	LUC-like 2		−1.937	0.0666
1439122_at		Ddx6	DEAD (Asp-Glu-Ala-Asp) box polypeptide 6		−1.819	0.0512
1427638_at		Zbtb16	zinc finger and BTB domain containing 16		−1.44	0.0903
1452899_at		Rian	RNA imprinted and accumulated in nucleus		−1.405	0.0512
1444228_s_at		Herc2	hect (homologous to the E6-AP (UBE3A) carboxyl terminus) domain and RCC1 (CHC1)-like domain (RLD) 2		−1.365	0.0903
1459619_at		Epb4.1l2	erythrocyte protein band 4.1-like 2		−1.365	0.0512
1449188_at		Midn	midnolin		−1.334	0.0512
1433804_at		Jak1	Janus kinase 1		−1.242	0.0903
1448352_at		Luzp1	Leucine zipper protein		−1.228	0.0512
1437422_at		Sema5a	semaphorin 5A		−1.237	0.0512
1443640_at		Zfp882	zinc finger protein 882		−1.203	0.0903
1443923_at		Akap13	A kinase (PRKA) anchor protein 13		−1.249	0.0512
1447787_x_at		Gjc1	gap junction protein, gamma 1		−1.179	0.0512

Transcripts that are upregulated in C57BL/6 mice with aging						
Affymetrix Probe Set ID	Gene Symbol			Gene Name	Fold Change	FDR (q value)
1425078_x_at	C130026I21Rik			RIKEN cDNA C130026I21 gene	2.945	0.0512
1437492_at	Mkx			mohawk homeobox	1.868	0.0512
1422873_at	Prg2			proteoglycan 2, bone marrow	1.845	0.0903
1435660_at	LOC664787			Sp110 nuclear body protein-like	1.676	0.0512
1422812_at	Cxcr6			chemokine (C-X-C motif) receptor 6	1.673	0.0512
1418608_at	Calml3			calmodulin-like 3	1.601	0.0903
1459030_at	Bbox1			butyrobetaine (gamma), 2-oxoglutarate dioxygenase 1 (gamma-butyrobetaine hydroxylase)	1.55	0.0512
1430567_at	Spink5			serine peptidase inhibitor, Kazal type 5	1.48	0.0512
1425832_a_at	Cxcr6			chemokine (C-X-C motif) receptor 6	1.442	0.0512
1457642_at	Skida1			SKI/DACH domain containing 1	1.428	0.0903
1426280_at	Wdpcp			WD repeat containing planar cell polarity effector	1.414	0.0666
1419528_at	Sult2a2			sulfotransferase family 2A, dehydroepiandrosterone (DHEA)-preferring, member 2	1.408	0.0512
1451840_at	Kcnip4			Kv channel interacting protein 4	1.399	0.0903
1427238_at	Fbxo15			F-box protein 15	1.348	0.0903
1453145_at	Pisd-ps3			phosphatidylserine decarboxylase, pseudogene 3	1.329	0.0512
1418871_a_at	Phgr1			proline/histidine/glycine-rich 1	1.345	0.0903
1455953_x_at	Pstk			phosphoseryl-tRNA kinase	1.299	0.0903
1418480_at	Ppbp			pro-platelet basic protein	1.273	0.0903
1439103_at	Cdc73			cell division cycle 73, Paf1/RNA polymerase II complex component	1.269	0.0512
1444040_at	Lair1			leukocyte-associated Ig-like receptor 1	1.265	0.0512
1425425_a_at	Wif1			Wnt inhibitory factor 1	1.26	0.0666

Similarly, we evaluated age-related molecular events in *Atp8b1* mutant lungs by transcriptome analysis of lungs at 7-9 (N=6) and 14 M (N=6) using Affymetrix Mouse 430v2.0 microarrays. Of the 532 genes that were differentially expressed in *Atp8b1* mutant lungs, there were 304 genes that were upregulated and 228 genes that were downregulated in an age-dependent manner (Figure 1A). The top differentially expressed genes (q<0.1, false discovery rate corrected Mann-Whitney U test p-value and median difference > 1.0), regardless of direction, are listed in (Table 2). Interestingly, the majority of gene transcripts were changed at least two-folds in either direction.

**Table 2:** Differentially expressed genes associated with aging in Atp8b1 mutant lungs (q<0.1, mean-value difference between two groups >1.0)

Transcripts that are upregulated in *Atp8b1* mutant mice with aging				
Affymetrix Probe Set ID		Gene Symbol		Gene Name	Fold Change	FDR
1418652_at		Cxcl9		chemokine (C-X-C motif) ligand 9	3.181	0.031
1419762_at		Ubd		ubiquitin D	3.161	0.031
1416957_at		Pou2af1		POU domain, class 2, associating factor 1	2.361	0.031
1438148_at		Cxcl3		chemokine (C-X-C motif) ligand 3	2.361	0.031
1418282_x_at		Serpina1b		serine (or cysteine) peptidase inhibitor, clade A, member 1B	2.359	0.031
1447792_x_at		Gpr174		G protein-coupled receptor 174	2.304	0.0546
1450912_at		Ms4a1		membrane-spanning 4-domains, subfamily A, member 1	2.236	0.0399
1423226_at		Ms4a1		membrane-spanning 4-domains, subfamily A, member 1	2.028	0.0399
1417851_at		Cxcl13		chemokine (C-X-C motif) ligand 13	2.006	0.031
1424374_at		Gimap4		GTPase, IMAP family member 4	1.932	0.0721
1418480_at		Ppbp		pro-platelet basic protein	1.916	0.0721
1417256_at		Mmp13		matrix metallopeptidase 13	1.914	0.031
1425832_a_at		Cxcr6		chemokine (C-X-C motif) receptor 6	1.893	0.031
1427221_at		Slc6a20a		solute carrier family 6 (neurotransmitter transporter), member 20A	1.868	0.031
1422978_at		Cybb		cytochrome b-245, beta polypeptide	1.855	0.0721
1422029_at		Ccl20		chemokine (C-C motif) ligand 20	1.851	0.0399
1422837_at		Scel		sciellin	1.764	0.0721
1425086_a_at		Slamf6		SLAM family member 6	1.751	0.0399
1422812_at		Cxcr6		chemokine (C-X-C motif) receptor 6	1.742	0.031
1436576_at		Fam26f		family with sequence similarity 26, member F	1.733	0.031
1454157_a_at		Pla2g2d		phospholipase A2, group IID	1.694	0.031
1425289_a_at		Cr2		complement receptor 2	1.666	0.031
1439141_at		Gpr18		G protein-coupled receptor 18	1.637	0.0399
1418776_at		Gbp8		guanylate-binding protein 8	1.634	0.031
1451513_x_at		Serpina1b		serine (or cysteine) preptidase inhibitor, clade A, member 1B	1.631	0.031
1448898_at		Ccl9		chemokine (C-C motif) ligand 9	1.612	0.0399
1451563_at		Emr4		EGF-like module containing, mucin-like, hormone receptor-like sequence 4	1.595	0.0947
1436649_at		Ikzf3		IKAROS family zinc finger 3	1.592	0.031
1449254_at		Spp1		secreted phosphoprotein 1	1.59	0.031
1425084_at		Gimap7		GTPase, IMAP family member 7	1.58	0.031
1419560_at		Lipc		lipase, hepatic	1.557	0.031
1444487_at		Lrat		lecithin-retinol acyltransferase (phosphatidylcholine-retinol-O-acyltransferase)	1.521	0.0546
1418826_at		Ms4a6b		membrane-spanning 4-domains, subfamily A, member 6B	1.49	0.031
1416318_at		Serpinb1a		serine (or cysteine) peptidase inhibitor, clade B, member 1a	1.454	0.0399
1421098_at		Stap1		signal transducing adaptor family member 1	1.453	0.0399
1448961_at		Plscr2		phospholipid scramblase 2	1.444	0.0721
1454159_a_at		Igfbp2		insulin-like growth factor binding protein 2	1.444	0.0399
1424375_s_at		Gimap4		GTPase, IMAP family member 4	1.413	0.031
1436941_at		Nxpe3		neurexophilin and PC-esterase domain family, member 3	1.41	0.0546
1418930_at		Cxcl10		chemokine (C-X-C motif) ligand 10	1.405	0.031
1449393_at		Sh2d1a		SH2 domain protein 1A	1.39	0.0721
1419728_at		Cxcl5		chemokine (C-X-C motif) ligand 5	1.385	0.0947
1417620_at		Rac2		RAS-related C3 botulinum substrate 2	1.383	0.0721
1422122_at		Fcer2a		Fc receptor, IgE, low affinity II, alpha polypeptide	1.382	0.0399
1424923_at		Serpina3g		serine (or cysteine) peptidase inhibitor, clade A, member 3G	1.379	0.031
1419598_at		Ms4a6d		membrane-spanning 4-domains, subfamily A, member 6D	1.373	0.0721
1423467_at		Ms4a4b		membrane-spanning 4-domains, subfamily A, member 4B	1.361	0.0721
1449373_at		Dnajc3		DnaJ (Hsp40) homolog, subfamily C, member 3	1.354	0.0947
1436779_at		Cybb		cytochrome b-245, beta polypeptide	1.346	0.0721
1436778_at		Cybb		cytochrome b-245, beta polypeptide	1.345	0.0399
1460273_a_at		Naip2		NLR family, apoptosis inhibitory protein 2	1.248	0.031
1449175_at		Gpr65		G-protein coupled receptor 65	1.247	0.031
1450632_at		Rhoa		ras homolog gene family, member A	1.24	0.0721
1417292_at		Ifi47		interferon gamma inducible protein 47	1.231	0.0399
1421168_at		Abcg3		ATP-binding cassette, sub-family G (WHITE), member 3	1.228	0.031
1420442_at		Cacna1s		calcium channel, voltage-dependent, L type, alpha 1S subunit	1.219	0.0546
1423182_at		Tnfrsf13b		tumor necrosis factor receptor superfamily, member 13b	1.212	0.0399
1439103_at		Cdc73		cell division cycle 73, Paf1/RNA polymerase II complex component	1.203	0.031

Transcripts that are downregulated in *Atp8b1* mutant mice with aging						
Affymetrix Probe Set ID	Gene Symbol		Gene Name		Fold Change	FDR
1416306_at	Clca3		chloride channel calcium activated 3		−3.201	0.0947
1439122_at	Ddx6		DEAD (Asp-Glu-Ala-Asp) box polypeptide 6		−2.153	0.031
1456379_x_at	Dner		delta/notch-like EGF-related receptor		−2.066	0.031
1455494_at	Col1a1		collagen, type I, alpha 1		−1.924	0.031
1449015_at	Retnla		resistin like alpha		−1.893	0.0721
1435990_at	Adamts2		a disintegrin-like and metallopeptidase (reprolysin type) with thrombospondin type 1 motif, 2		−1.829	0.031
1433757_a_at	Nisch		nischarin		−1.825	0.0399
1447853_x_at	Kif13a		kinesin family member 13A		−1.811	0.031
1428936_at	Atp2b1		ATPase, Ca++ transporting, plasma membrane 1		−1.792	0.031
1423065_at	Dnmt3a		DNA methyltransferase 3A		−1.714	0.0399
1424598_at	Ddx6		DEAD (Asp-Glu-Ala-Asp) box polypeptide 6		−1.648	0.031
1420402_at	Atp2b2		ATPase, Ca++ transporting, plasma membrane 2		−1.544	0.031
1456102_a_at	Cul5		cullin 5		−1.536	0.0721
1456489_at	Pcf11		cleavage and polyadenylation factor subunit homolog (S. cerevisiae)		−1.535	0.031
1438069_a_at	Rbm5		RNA binding motif protein 5		−1.509	0.0721
1460069_at	Smc6		structural maintenance of chromosomes 6		−1.494	0.0399
1436746_at	Wnk1		WNK lysine deficient protein kinase 1		−1.478	0.0721
1444547_at	Ankrd40		ankyrin repeat domain 40		−1.477	0.031
1423669_at	Col1a1		collagen, type I, alpha 1		−1.476	0.031
1423110_at	Col1a2		collagen, type I, alpha 2		−1.446	0.031
1415994_at	Cyp2e1		cytochrome P450, family 2, subfamily e, polypeptide 1		−1.443	0.0399
1438726_at	Mical2		microtubule associated monooxygenase, calponin and LIM domain containing 2		−1.421	0.0546
1427884_at	Col3a1		collagen, type III, alpha 1		−1.416	0.031
1442878_at	Prdx6		peroxiredoxin 6		−1.411	0.031
1427768_s_at	Myl3		myosin, light polypeptide 3		−1.4	0.0721
1458439_a_at	Dzip3		DAZ interacting protein 3, zinc finger		−1.323	0.0399
1426287_at	Atxn7		ataxin 7		−1.319	0.031
1452899_at	Rian		RNA imprinted and accumulated in nucleus		−1.317	0.0947
1437422_at	Sema5a		sema domain, seven thrombospondin repeats (type 1 and type 1-like), transmembrane domain (TM) and short cytoplasmic domain, (semaphorin) 5A		−1.316	0.031
1452378_at	Malat1		metastasis associated lung adenocarcinoma transcript 1 (non-coding RNA)		−1.313	0.0399
1459804_at	Crebbp		CREB binding protein		−1.248	0.031
1433758_at	Nisch		nischarin		−1.241	0.031
1438403_s_at	Malat1 /// Ramp2		metastasis associated lung adenocarcinoma transcript 1 (non-coding RNA) /// receptor (calcitonin) activity modifying protein 2		−1.241	0.0947
1438083_at	Hhip		Hedgehog-interacting protein		−1.232	0.031
1457193_at	Kmt2c		lysine (K)-specific methyltransferase 2C		−1.223	0.0399
1427883_a_at	Col3a1		collagen, type III, alpha 1		−1.213	0.031
1447863_s_at	Nr4a2		nuclear receptor subfamily 4, group A, member 2		−1.21	0.0399
1452360_a_at	Kdm5a		lysine (K)-specific demethylase 5A		−1.199	0.031
1455277_at	Hhip		Hedgehog-interacting protein		−1.196	0.031
1437933_at	Hhip		Hedgehog-interacting protein		−1.167	0.031
1418562_at	Sf3b1		splicing factor 3b, subunit 1		−1.117	0.031

Next, we compared the gene profiling dataset from *Atp8b1* mutant and C57BL/6 mice to determine the variance in gene transcripts between normal aging and aging with the gene mutation. We found 37 genes that overlapped between the two datasets, 350 genes unique to the *Atp8b1* mutant, and 85 unique to C57BL/6 datasets (Figure 1B). Some of the transcripts that overlapped between C57BL/6 and *Atp8b1* mutant lungs were found to decrease with aging. These included *Akap13*, *Atrx*, *Col1a1*, *Col3a1*, *Ddx6, Luzp1, Rian*, *Sema5a*, and *Sfb31*, whereas transcripts such as *Cxcr6 and Slc6A20* increased with aging (Table S1). Notably, transcripts *Col1a1* and *Col3a1* encoding collagen were expected to decrease with age based on previous literature [19].

### IPA identifies key gene networks in aged C57BL/6 lungs

**Figure 2:**
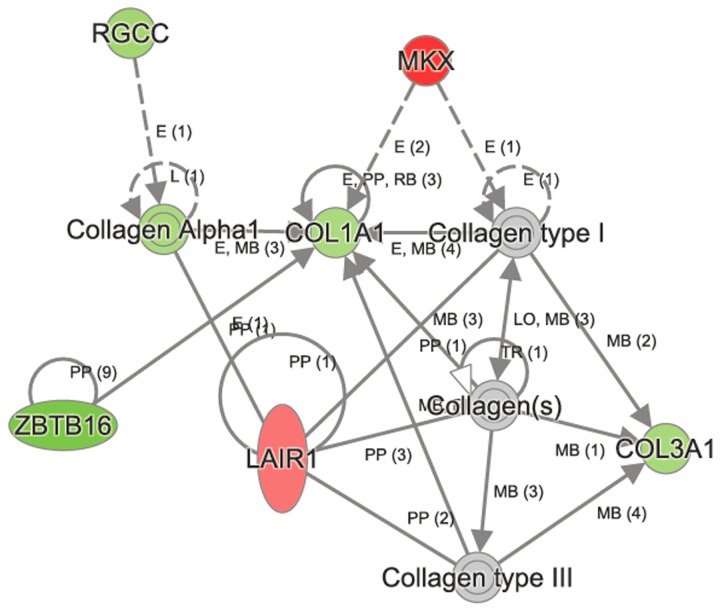
Ingenuity Pathway Analysis (IPA) identified perturbation of cellular assembly, organization, function, and maintenance in aged network to be perturbed in aged C57BL/6 lungs Some of the key molecules in this network are shown in the figure. The genes *Col1a*, *Col3a1*, *Zbtb16* and *Rgcc* were downregulated, whereas *Mkx* and *Lair* were upregulated in aged C57BL/6 lungs.

To identify the gene networks and biological pathways that are perturbed in normal aging, we examined the microarray data from C57BL/6 lungs by Ingenuity Pathway Analysis (IPA). The top canonical pathways (q<0.05) that were altered significantly in C57BL/6 lungs in age-dependent manner were vitamin C transport, intrinsic prothrombin activating pathway, hematopoiesis, from pluripotent stem cells, L-carnitine biosynthesis and ascorbate recycling (Table 3). Other canonical pathways that changed significantly in C57BL/6 were RhoA, PI3K/Akt, Oncostatin M, EGF, PDGF, Jak/Stat, iNOS, and Wnt/ β-catenin signaling pathways (Table S2). The upstream regulators of these canonical pathways included CMA1, MKL1, MKL2, NR4A2, and miR-296. One of the networks affected in C57BL/6 aging process was “cellular assembly, organization, function, and maintenance,” and includes 38 molecules (Figure 2). Some of the key molecules in this network are *Akap13* (−1.249), *Col1a1* (−1.042), *Col3a1* (−1.071), *Gjcl* (−1.179)*, Jak1* (−1.249)*, Lair* (1.265)*, Mkx* (1.868)*, Rgcc* (−1.153), and *Zbtb16* (−2.0).

**Table 3:** Canonical pathways identified in aged C57BL/6 and *Atp8b1* mutant lungs

**Name**	**Molecules**	**-log(p-value)**
**Top Canonical Pathways in C57BL/6 Mice**		
		
Vitamin-C Transport	GSTO2,SLC23A1	2.48E00
Intrinsic Prothrombin Activation Pathway	COL3A1,COL1A1	1.91E00
Hematopoiesis from Pluripotent Stem Cells	IGHM,IGHA1	1.91E00
L-carnitine Biosynthesis	BBOX1	1.73E00
Ascorbate Recycling (Cytosolic)	GSTO2	1.73E00
		
**Top Canonical Pathways in *Atp8b1* Mice**		
**Name**	**Molecules**	**-log(p-value)**
OX40 Signaling Pathway	HLA-A,CD3G,HLA-DQA1,HLA-DQB1,CD3D,HLA-DMB,HLA-DOB,B2M,NFKBIE	6.25E00
Altered T Cell and B Cell Signaling in Rheumatoid Arthritis	SLAMF1,TNFRSF17,CD28,CXCL13,HLA-DQA1,TNFRSF13B,HLA-DQB1,CD79B,	6.06E00
	HLA-DMB,HLA-DOB,SPP1	
Communication between Innate and Adaptive Immune Cells	TNFRSF17,CD28,HLA-A,CD8A,CCL5,CXCL10,TNFRSF13B,Ccl9,B2M,IGHA1	6.06E00
Antigen Presentation Pathway	HLA-A,CD74,HLA-DQA1,HLA-DMB,HLA-DOB,B2M,PSMB8	
Agranulocyte Adhesion and Diapedesis	CCXL9,CCL8,CCL5,CXCL10,MMP12,CCL20, MMP13,Glycam1,Ppbp,CXCL6,CCL19, CXCL13, CCL9, CXCL3,MYL3	5.98E00

**Figure 3:**
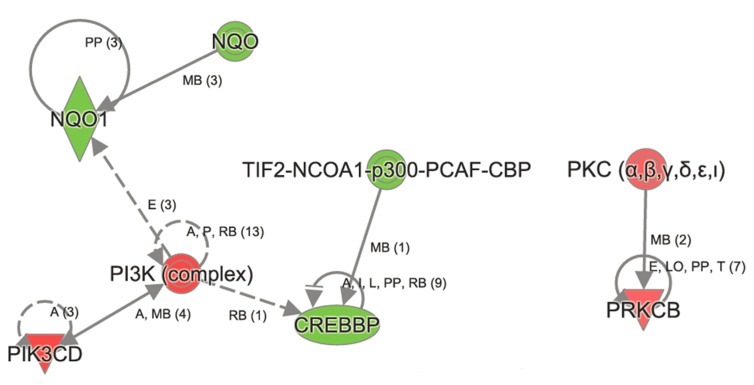
Ingenuity Pathway Analysis revealed genes in Xenobiotic metabolism to be affected in aged *Atp8b1* mutant mice One of the most important transcript in Xenobiotic metabolism namely *NQO1* was decreased in aged *Atp8b1* mutant lungs. Members that were decreased in Xenobiotic metabolism included CREBBP and p300. The transcript encoding members in PI3K complex (PIKCD) and PKC α,β (PRKCB) were increased in aged *Atp8b1* mutant lungs.

**Figure 4:**
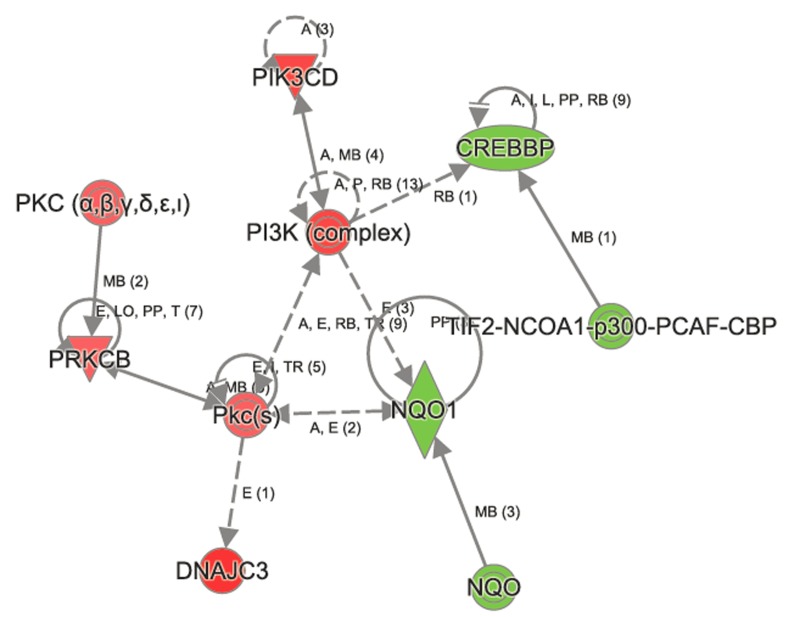
Ingenuity Pathway Analysis shows perturbation in Nrf2 signaling in aged *Atp8b1* mutant mice The transcripts encoding NQO1, CREBBP, p300 were decreased in aged *Atp8b1* mutant mice. Members in PI3K complex (PIK3D), PKC α,β (PRKCB, PKc(s) and DNAJC3 were increased in aged Atp8b1 mutant lung.

**Figure 5:**
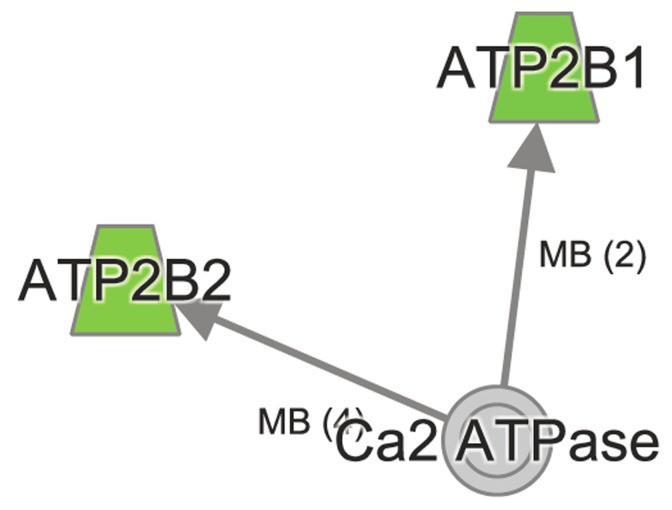
Ingenuity Pathway Analysis reveals dys-regulated Calcium signaling in aged *Atp8b1* mutant mice The transcripts *Atp2b1* and *Atp2b2* encoding ATP2B1 and ATP2B2 that play an important role in calcium signaling were decreased in *Atp8b1* mutant lung in age-dependent manner.

**Figure 6:**
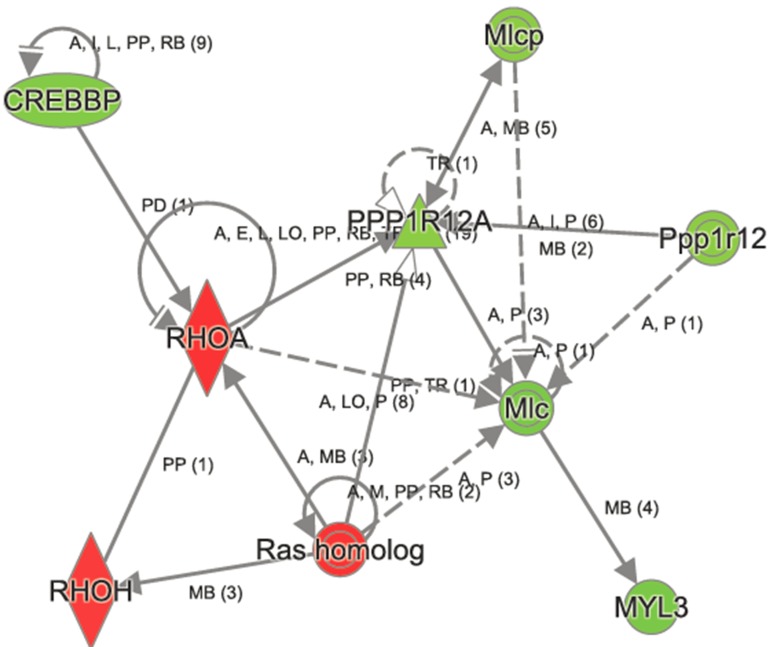
Ingenuity Pathway Analysis of RhoGD1 signaling in aged *Atp8b1* mutant mice The transcript encoding key molecules in this pathway, namely, RHOA, RHOH and Ras homologs were increased, whereas CREBBP, MYL3, Mlcp, and Ppp1r12 were decreased in aged *Atp8b1* mutant mice. Symbols and Color Keys. In the Figures (2-6), upregulated genes are depicted in red and downregulated genes in green. The solid lines depict direct interaction and the dashed lines depict indirect interaction between genes. The arrow represents interaction between genes. The symbols represent the following. A= Activation, B= Binding, E= Expression, I= Inhibition, PP = Protein-Protein binding, P = Phosphorylation/Dephosphorylation, RB= regulation of binding, MB = Group/Complex membership.

In contrast, the top canonical pathways significantly changed in *Atp8b1* mutant as a result of aging are OX40 signaling, altered T cell and B cell signaling in rheumatoid arthritis, communication between innate and adaptive immune cells, the antigen presentation pathway, agranulocyte adhesion, and diapedesis (Table 3). Other canonical pathways that were significantly altered in *Atp8b1* mutant mice attributed to aging were: calcium, RhoA and Rac signaling, and the xenobiotic metabolism (Table S3). The important molecules in xenobiotic metabolism and Nrf2 signaling are *Nqo1* (−1.252), *Crebbp* (−1.248), and *Pik3cd* (1.219). The signaling molecules in RhoGD1 pathway are *RhoA* (1.241) and *Rhoh* (1.174) (Figure 6).

Interestingly, the metalloproteinase family members *Mmp12* and *Mmp13* were upregulated in *Atp8b1* mutant mice relative to C57BL/6 mice in an age-dependent manner. Further, many of genes linked to collagen production in lungs such as *Col1a1*, *Col1a2*, *Col3a1*, and *Col5a1* were downregulated with age in *Atp8b1* mutant mice. *Adamts2* encoding a disintegrin and metalloproteinase with thrombospondin Type 1 Motif, 2 was also downregulated in *Atp8b1* mutant mice in age-dependent manner. In contrast, *Serpina 1b* encoding alpha-1 anti-trypsin was upregulated with age in *Atp8b1* mutant mice suggesting its role in protecting lung tissue against neutrophil elastase. Interestingly, NAD(P)H dehydrogenase quinone 1 (NQO1) transcript, a Nrf2 target gene was significantly decreased in aged Atp8b1 mutant lungs. Another anti-oxidant gene, *Prdx6* encoding peroxiredoxin 6, involved in reducing phospholipid hydroperoxide, was also similarly decreased in *Atp8b1* mutant lungs.

### Quantitative Real-time PCR analysis for differentially expressed transcripts in C57BL/6 and *Atp8b1* mutant mice

**Figure 7:**
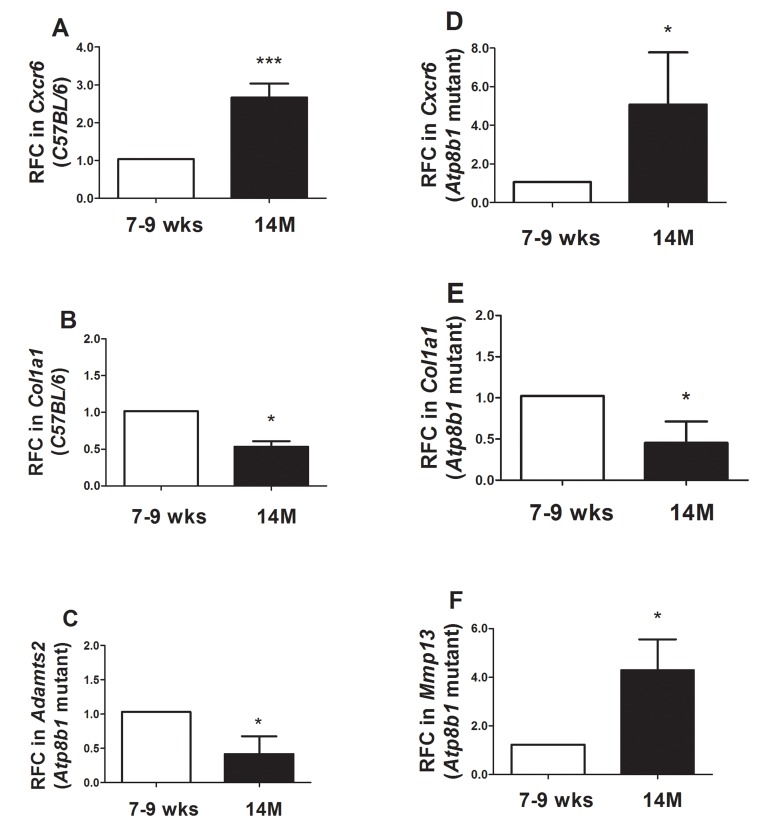
Quantitative Real-time RT-PCR analysis confirmed several transcripts in *Atp8b1* mutant and C57BL/6 lungs at 7-9 wks vs. 14 M (**A**) In C57BL/6 mice, *CxCr6* transcript increased significantly at 14M relative to 7-9 wks.*** p < 0.001 relative to 7-9 wks time point. (**B**) In C57BL/6 mice, *Col1a1* transcript significantly decreased at 14M compared to 7-9 wks. * p < 0.05 relative to 7-9 wks time point. (**C**) In *Atp8b1* mutant, *Adamts2* was significantly decreased at 14M when compared to 7-9 wks. * p < 0.05 relative to 7-9 wks time point. (**D**) *CxCr6* transcript was significantly increased in 14M *Atp8b1* mutant lungs versus 7-9 wks *Atp8b1* mutant lungs. * p < 0.05 relative to 7-9 wks time point. (**E**) *Col1a1* transcript levels decreased significantly at 14M relative to 7-9 wks *in Atp8b1* mutant lungs. * p < 0.05 relative to 7-9 wks time point. (**F**) *Mmp13* was significantly increased in aged *Atp8b1* mutant lungs versus young adult lungs (7-9 wks). * p < 0.05 relative to 7-9 wks time point. For the qRT-PCR, N=6 mice were tested per group. Student's T test was used to calculate statistical significance between the two groups.

Some transcripts that were decreased or increased in *Atp8b1* mutant lungs during aging were independently validated using qRT-PCR analysis. *Cxcr6* encoding chemokine receptor 6 that was significantly increased in C57BL/6 during aging was validated (Figure 7A).

*Col1a1* which was decreased in C57BL/6 mice in aging was also validated by qRT-PCR (Figure 7B). *Adamts2* encoding an ADAM metallopeptidase with thrombo-spondin Type 1 Motif, 2 was also validated by qRT-PCR analysis (Figure 7C). Similar to C57BL/6 mice, an increase in *CxCr6* transcript encoding chemokine receptor 6 in *Atp8b1* mutant mice was validated (Figure 7D). Likewise, *Col1a1* was significantly decreased at 14 M relative to 7-9 wks in *Atp8b1* mutant lungs and validated our microarray findings (Figure 7E). Lastly, a 4-fold increase in *Mmp13* transcript was observed at 14 M in *Atp8b1* mutant lungs (Figure 7F).

### Nrf2 protein levels are decreased in aged *Atp8b1* mutant lungs

**Figure 8:**
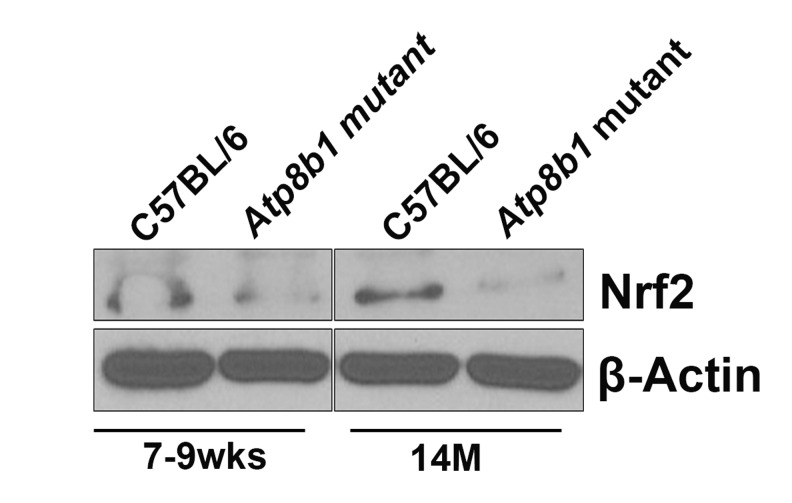
Western blot analysis of Nrf2 Equal amounts of protein from C57BL/6 and *Atp8b1* mutant lung homogenates (7-9 wks and 14 M timepoints) were separated on 10% SDS-PAGE and probed with anti-Nrf2 antibody. There was no change in Nrf2 protein levels at 7-9 wks in both the groups. There was a significant decrease in Nrf2 protein in *Atp8b1* mutant lungs at 14 M compared to C57BL/6 control. This is a representative blot. The experiment was repeated three times. β–Actin used as a loading control shows equal loading in all the lanes.

Based on our microarray data, antioxidant Nrf2 target gene, NAD(P)H dehydrogenase, quinone 1 (NQO1) transcript was decreased significantly in aged *Atp8b1* mutant lungs. As we were interested in exploring the Nrf2 pathway in detail, we carried out Western blot analysis of Nrf2 protein and detected a 110 kDa protein as described [20]. We found that at 7-9 wks, there was no change in Nrf2 protein in C57BL/6 or *Atp8b1* mutant lungs (Figure 8). Interestingly at 14 M, in *Atp8b1* mutant lungs, there was a decrease in Nrf2 protein relative to C57BL/6 control (Figure 8).

## DISCUSSION

Mitochondrial dysfunction is associated with aging [9]. Cardiolipin, a phospholipid found in the inner mito-chondrial membrane, is vital for overall mitochondrial function and maintenance of membrane potential and integrity. In many pathological conditions associated with aging, there is alteration in the structure and content of cardiolipin leading to mitochondrial dysfunction [11]. Atp8b1 is a cardiolipin transporter in type II AECs [15]. The G308V mutation leads to a functional deficiency in Atp8b1 via aberrant folding of Atp8b1 in the endoplasmic reticulum (ER) and decreased expression of Atp8b1 in the plasma membrane [15, 21]. Therefore, Atp8b1 mutant proteins expressed in type II AECs are defective in cardiolipin transport. Further, mutation in *Atp8b1* or lung inflammation is associated with increased secretion of cardiolipin in airways and also development of pneumonia [15]. In this study, we examined age-related genes by global transcriptome analysis of lung tissue derived from *Atp8b1* mutant and C57BL/6 mice. Studies from our lab indicate that *Atp8b1* mutant mice are more susceptible to oxidative stress-induced lung injury during hyperoxia (unpublished observations). Therefore, we were interested in studying the transcriptome of *Atp8b1* mutant mice at 7-9 wks and 14 M to elucidate age-related changes that contribute to disease susceptibility.

Comparison of *Atp8b1* mutant lungs at 7-9 wk and 14 M revealed 537 differentially expressed genes relative to the 157 observed in the C57BL/6 lung samples. Of these, 37 genes overlapped between *Atp8b1* mutant and C57BL/6 lung samples indicating common age-related genes between these genotypes. The overlapping genes including *Akap13*, *Atrx*, *Col1a1*, *Col3a1*, *Ddx6*, *Luzp1*, *Rian*, *Sema5a*, and *Sfb31,* which decreased with aging; whereas transcripts including *Cxcr6* and *Slc6A20* increased with aging. In both C57BL/6 and *Atp8b1* mutants, collagen producing genes such as *Col1a1*, *Col1a2*, *Col3a1*, and *Col5a1* were decreased during aging. These findings are in agreement with a study wherein age-related changes in collagen production and degradation were observed in aging rat lungs [19].

One of the strengths of our study is that we used *Atp8b1* mutant mice on C57BL/6 background and compared them to C57BL/6 normal controls. The effect of mouse strain background in aged lungs has previously revealed some major differences between C57BL/6 and DBA/2 mice [9]. There was an up-regulation of stress-related genes including xenobiotic detoxification system in DBA/2 mice; whereas in C57BL/6 mice, there was a down-regulation of heat-shock genes. There was also an age-dependent down-regulation of collagen genes in both strains [9]. Gene profiling studies in various aging mouse models reveal up-regulation of stress-response genes including heat-shock response and oxidative-stress inducible genes [8, 11].

Furthermore, a majority of gene changes were observed only in *Atp8b1* mutant lungs relative to the C57BL/6 controls and may be attributed to both the effect of mutation and aging (14 M). IPA analysis revealed distinct changes in the canonical and non-canonical signaling pathways in aged *Atp8b1* mutant mice relative to the aged C57BL/6 controls. Some of the important canonical pathways altered in *Atp8b1* mutants included xenobiotic metabolism, Nrf2, calcium, and RhoGD1 signaling. The majority of the signaling molecules in these pathways were decreased in *Atp8b1* mutant lungs. In contrast, some of the canonical pathways altered in C57BL/6 were: RhoA, PI3K/Akt, EGF, PDGF, Jak/Stat, iNOS, and Wnt/β-catenin signaling. The RhoA pathway was altered in both *Atp8b1* mutant and C57BL/6 lungs during aging. RhoA is an important signaling molecule that plays a pivotal role in cell proliferation, growth, adhesion, and also calcium sensitivity. An age related increase in both *RhoA* transcript and protein content was observed in the aortic and basilar arteries in rats [22]. This suggests that RhoA may play an important role in vascular responses associated with aging including: injury and/or cell proliferation and migration. Besides, RhoA is known to regulate the actin cytoskeleton and is involved in cell cycle progression and gene regulation [23]. Previously, it has been demonstrated that Rho signaling mediates both growth factor expression in fibroblasts and formation of myofibroblast in IPF lungs [24, 25]. The importance of RhoA signaling in modulating Cyclin D1 expression in IPF-derived fibroblasts and its effect on fibroblast proliferation has also been reported [26]. Moreover, age-related diseases, such as IPF, may share some common pathophysio-logical mechanism with normal lung aging [1, 27, 28]. Therefore, it is plausible that an increase in *RhoA* transcript in both aged *Atp8b1* mutant and aged C57BL/6 mice may be associated with response against cell injury, proliferation, or migration.

In addition, *Rac* and *Rho* transcripts were significantly increased in aged *Atp8b1* mutant mice. Rac and Rho proteins are involved in non-canonical Wnt signaling pathway. In contrast, the gene encoding cAMP-response element binding protein (CREBP), which forms a complex with β-catenin and acts as a transcriptional co-activator in canonical Wnt signaling pathway, was significantly decreased in *Atp8b1* mutant mice in an age-dependent manner. Wnt signaling pathways have been associated with replicative senescence that is one of the hallmarks of aging [29]. Our observation that canonical Wnt signaling decreased in *Atp8b1* mutant lungs is in concordance with another study wherein a decrease in *Tle1* and *Lef1* transcripts and increase in *Frzb*, encoding an extracellular Wnt ligand inhibitor, were observed in 24 M lungs relative to 5 M lungs [30]. The decline of Wnt signaling has been associated with several age-related diseases such as Alzheimer's disease, Parkinson's disease [31], and osteoporosis [32, 33]. This may be attributed to the important role of Wnt signaling in the maintenance of stem cell homeostasis and delay of aging. On the other hand, over activation of Wnt signaling has been reported to result in tissue fibrosis [34]. Based on our data and the previous report [30], we observe a decline in the Wnt pathway.

In addition*, Adamts2*, a transcript encoding a disintegrin metalloprotease with thrombospondin type 1 motif, 2 linked to ECM, was found to be decreased in aged *Atp8b1* mutant lungs. This is in agreement with another study where genes encoding collagen and ECM showed a decrease with age in both wild type mouse strains (DBA/2J and C57BL6/J) [35,19]. Matrix metallo-proteinases (MMPs) play an important role in fibrosis and tissue remodeling. MMPs regulate deposition and resorption of collagen and other ECM components. The turnover of ECM is determined by the ratio of MMPs and the tissue inhibitor of matrix metalloproteinases (TIMPs) [36]. We speculate that in *Atp8b1* mutant mice, the age-dependent reduction in collagen isoforms may be linked to the aberrant re-modeling of ECM by MMPs and TIMPs. Further, the transcriptome profile of *Atp8b1* mutant lungs revealed increased expression of *Mmp12* and *Mmp13* mRNAs that encode metalloproteinases MMP12 and MMP13, respectively. MMP12 is a macrophage-specific metallo-proteinase that specifically degrades elastin and has been associated with chronic degenerative emphysema [37]. In addition, the gene profiling of mice that lack integrin b6 subunit of the αvβ6 integrin showed an 18-fold increase in *Mmp12* [38]. The deficiency of integrin αvβ6-mediated TGF-β activation leads the mice to spontaneously develop emphysema at 14M of age in a MMP12 dependent manner [38]. In light of this finding, *Atp8b1* mutant mice may be susceptible to develop age-induced emphysema as a result of increased *MMP12* levels. Interestingly, in an asbestos-induced mouse model of lung injury, the collagenase MMP13 has been shown to play a key role in the development of severe inflammation and fibrosis [39]. Moreover, MMP13 knockout mice have been shown to exhibit reduced radiation-mediated inflammation and fibrosis [40]. Interestingly, MMP13 is highly upregulated in IPF patient lungs and its spatially imbalanced collagenolytic activity in airways results in the development of characteristic honeycomb cysts [6]. This suggests that *Atp8b1* mutant mice may be susceptible to age-induced lung fibrosis mediated in part by MMP13.

In a Schistosoma mansoni model of pulmonary fibrosis, Madala and colleagues demonstrate the intricate balance between MMP12 and MMP13 in modulating TH2-dependent lung and liver fibrosis [41]. They show that in MMP12 ^−/−^ mice, there was a marked increase of MMP13 after *S. mansoni* egg challenge. In addition, they show that MMP12 may negatively regulate MMP13 expression in liver and lung. Further, MMP12 expression was dependent upon IL-4/IL-13 signaling [41]. It would be interesting to explore IL-4 and IL-13 signaling in our *Atp8b1* mutant mouse model as well as check whether TH2 predominates over TH1 response in our model. Though the TGF-β signaling pathway is implicated in lung fibrosis, in their model of parasite-induced fibrosis, TGF-β signaling was not implicated. In concordance with this and previous studies, we did not observe any transcriptome changes in TGF-β signaling with aging in either C57BL/6 or *Atp8b1* mutant mice [34].

Excessive production of ROS has been linked to age-related diseases such as COPD, IPF, and lung fibrosis [3, 4, 6]. ROS-mediated accelerated aging also induces chronic inflammation in COPD lungs [4]. The inflammation associated with COPD or other chronic inflammatory lung diseases is attributed to the loss of alpha1-antitrypsin function [42]. Interestingly, our gene profiling studies showed that the expression of *Serpina1b*, a gene that encodes for alpha1-anti-trypsin was up-regulated in aged *Atp8b1* mutant mice. Further, alpha-1 antitrypsin is known to protect the lungs from neutrophil elastase. This indicates that the susceptibility of *Atp8b1* mutant mice to inflammatory or age-related lung disease is not attributed to deficiency of alpha1-antitrypsin.

In addition, several endogenous antioxidant mechanisms are present to combat ROS-mediated damage in cells including the Nuclear factor erythroid-2-related factor 2-antioxidant response element (Nrf2-ARE) pathway [5]. Antioxidant response element (ARE) is a cis-acting element found in the promoter region of several cytoprotective genes including GSTA2 (glutathione S-transferase A2) and NQO1 (NADPH: quinone oxidoreductase 1) [43]. The unique responsiveness to oxidative stress, changes in redox potential and elevated electrophilic species is a structural and biological feature of the ARE [44]. The transcription factor Nrf2 is a positive regulator of ARE and is expressed in epithelial and alveolar macrophages as it plays a vital role in protecting lungs from ROS-mediated injury via activation of ARE-induced cytoprotective genes [5]. The protective effect of Nrf2 against oxidative stress is supported by studies that show an increased incidence of cancer, pulmonary disease, and inflammation in Nrf2 knockout mice (reviewed in [45-47]). Interestingly, Nrf2 signaling plays a protective role in many of these age-relative diseases. Nrf2 protects against the development of pulmonary fibrosis by regulating the redox level in the cells and by maintaining Th1/Th2 balance [48]. In another related study, the targeting of the Nox4-Nrf2 pathway to restore the Nox4-Nrf2 redox balance in myofibroblasts was suggested as an effective therapeutic strategy in age-dependent fibrotic disorders [27]. In our study, we found that one such Nrf2 target gene, *Nqo1* encoding NAD(P)H dehydrogenase, quinone 1 (NQO1). was significantly decreased in aged *Atp8b1* mutant lungs relative to control. This suggests that the Nrf2 anti-oxidant pathway may be perturbed in *Atp8b1* mutant mice. It is plausible that this may account for the oxidative stress induced phenotype observed when these mice were treated with hyperoxia. Another anti-oxidant gene, *Prdx6* encoding peroxiredoxin 6, involved in reducing phospholipid hydroperoxide was similarly decreased in *Atp8b1* mutant lungs. PRDX6 protein is involved in redox regulation and protection of cells against oxidative injury in an ARE-dependent manner via the Nrf2 pathway.

In conclusion, our transcriptome analysis revealed distinct signaling pathways such as RhoA and Wnt, shown to be perturbed in aged lungs in both C57BL/6 and *Atp8b1* mutant mice. In addition, collagen producing genes such as *Col1a1*, *Col1a2*, *Col3a*, and *Col5a1* were also decreased during aging in both C57BL/6 and *Atp8b1* mutant mice. In addition, we observed age-related changes in *Atp8b1* mutant mice transcriptome including *Mmp12*, *Mmp13*, *Adamts2*, *Nrf2* signaling and xenobiotic signaling pathways. This suggests that some of the signaling pathways commonly affected in C57BL/6 and *Atp8b1* mutant mice can be attributed to the process of aging, whereas the transcripts affected only in *Atp8b1* mutant mice with aging could be due to the combined effect of the *Atp8b1* mutation and the aging process. Future studies will explore in greater detail whether *Atp8b1* mutant mice are susceptible to age-related lung fibrosis and the molecular mechanisms underlying the disease processes.

## METHODS

### Ethics statement

Protocols using mice in this study were approved by the University of South Florida Institutional Animal Care and Use Committee, IACUC (Animal Welfare Assurance Number: A4100-01) in accordance with the “Guide for the Care and Use of Experimental Animals” established by the National Institute of Health (NIH) (1996, Revised 2011).

### Animals

*Atp8b1^G308V/G308V^* mutant mouse was a generous gift from Dr. Laura Bull (University of California, San Francisco). C57BL/6 mice were purchased from Harlan laboratories (Indianapolis, IN) and were used as controls. *Atp8b1^G308V/G308V^* (N=6, 7-9 weeks old males; N=6, 7-9 weeks old females; and C57BL/6 (N=6, 14M old males) and (N=6, 14M old females) were maintained in a specific-pathogen-free animal facility at the University of South Florida. We provided water and standard food *ad libitum*.

### Collection of mouse lungs

Mice were anesthetized with intraperitoneal injection of a ketamine/xylazine mixture. Following thoracotomy, the inferior vena cava (IVC) was clamped and 2 ml of sterile phosphate-buffered saline (PBS) was injected into the right ventricle for lung perfusion as previously described [2]. The lung samples were flash frozen in liquid nitrogen.

### Total RNA extraction

Total RNA from C57BL/6 and *Atp8b1* mutant lungs (7-9 wks and 14 M) was extracted using Trizol (Thermo Fischer Scientific, Tampa, FL) and RNeasy kit (Qiagen, Valencia, CA, USA) as per manufacturer's instructions with minor modifications Briefly, lungs were homogenized in Trizol. This was followed by the extraction of aqueous phase using chloroform and precipitation of RNA using isopropanol. The precipitated RNA was dissolved in 30 μl of RNase-free water and subjected to purification using RNeasy columns as per manufacturer's instructions (Qiagen). The quality of RNA was assessed in three ways, first by Agilent Bioanalyzer, second by testing for RNase activity and running samples on agarose gels, and third, by checking the absorbance of the total RNA by Nanodrop. RNA samples with 260/280 ratio of 1.8 to 2.0 and 260/230 ratio of > 2.0 were further considered for microarray analysis. The samples that passed all the three quality control (QC) tests were considered suitable for microarray processing.

### Transcriptome analysis of lungs

Mouse Genome 430 v2.0 arrays (Affymetrix) contains over 45,000 probe sets designed from GenBank, dbEST, and RefSeq sequences that were clustered based on build 107 of the UniGene database. The clusters were further refined by comparison to the publicly available draft assembly of the mouse genome. An estimated 39,000 distinct transcripts are detected including over 34,000 well substantiated mouse genes. Each gene is represented by a series of oligonucleotides that are identical to the sequence in the gene as well as oligonucleotides that contain ahomomeric (base transversion) mismatch at the central base position of the oligomer, which is used to measure cross-hybridization. Microarray study was performed at the Molecular Genomics Core (MGC) at Moffitt Cancer Center. A brief procedure is as described below.

### Sample processing and preparation of library

One hundred micrograms of total RNA was converted to cDNA, amplified and labeled with biotin using the Ambion Message Amp Premier RNA Amplification kit (Life Technologies, Grand Island, NY) as per manufacturer's protocol [49]. This was followed by hybridization with the biotin-labeled RNA, staining, and scanning of the chips as outlined in the Affymetrix technical manual and described previously [50].

### Data analysis

Scanned output files were visually inspected for hybridization artifacts and then analyzed using Affymetrix Expression Console 1.4 software using the MAS 5.0 algorithm. Signal intensity was scaled to an average intensity of 500 before data export and filtering [51]. The data was further analyzed at Cancer Informatics Core at Moffitt Cancer Center.

### Differential gene expression

The CEL files were normalized using pair-wise IRON (Iterative Rank-order normalization) method as described previously (http://gene.moffitt.org/libaffy) [52] and QC was checked using Sample-to-sample scatter plot and Principal Component Analysis (PCA) for all samples and all variables (Strain, time-points, and gender). No outlier or batch effects were detected. The first five samples were all males. One sample was removed from further analysis (B6 7-9wks, Male 6) since the expression of gender specific genes did not match its annotated gender. For each probe set, the raw intensities were log2-transformed. Significantly differently expressed genes was defined as q<0.1 (false discovery rate corrected Mann-Whitney U test p-value) and if the median-value difference between two groups was larger than 1 (log2 expression units). Differentially expressed genes were further analyzed using the Ingenuity Pathway Analysis, (IPA) (Ingenuity Systems, Qiagen). MIAME compliant microarray data was submitted to Gene expression omnibus (GEO) database (http://www.ncbi.nlm.nih.gov/geo) and the assigned GEO Accession Number is (GSE80680).

### Ingenuity Pathway Analysis (IPA)

Ingenuity Pathway Analysis software (IPA; Ingenuity Systems, www.ingenuity.com, Summer 2015 release, Qiagen) was used to identify gene networks affected in C57BL/6 and *Atp8b1* mutant mice at two different time-points (7-9 wk vs 14 M). Data that was uploaded into IPA included Affymetrix Mouse Genome 430 V 2.0 probe sets as identifiers as well as processed microarray data sets. Using Ingenuity Pathway Analysis, canonical pathways of differentially expressed genes that were most significant were identified. Only genes having q< 0.05 were used for analysis. Based on their connectivity to the uploaded data, gene networks were algorithmically generated using this software. In each network, the genes or molecules are represented as nodes, and the biological relationship between two nodes is represented as an edge (line). All edges are supported by at least one reference from the literature or canonical information stored in IPA knowledge base. The intensity of the node color indicates the degree of up- (red) or down- (green) -regulation with respect to the datasets. Nodes are displayed using various shapes that represent the functional class of the gene product.

### Quantitative Real-time RT-PCR (qRT-PCR) analysis

One microgram of total RNA was reverse transcribed using the iScript cDNA synthesis kit as per manufacturer's instructions (Biorad laboratories, Hercules, CA). We performed qRT-PCR with the SsoFast EvaGreen Supermix kit (Biorad) and gene-specific primers using the Biorad CFX96 Real-time system (C1000 Thermal Cycler). A relative fold change in gene transcript was calculated using the Biorad CFX Manager software, applying the comparative CT method (Delta C_T_) and expressed as 2^−ddCT^ and using β–actin as an internal calibrator. Melting curve analysis was performed to determine the specific amplification of the target genes. The primers for qRT-PCR were designed using the mouse qPrimerDepot (http://mouseprimerdepot.nci.nih.gov). The primers used for qRT-PCR are listed (Table 4).

**Table 4:** Quantitative Real-time PCR primers

Gene	Primers (5′-3′)	Product size (bp)
*ActB*	Forward: CCAGTTCGCCATGGATGACGATAT Reverse: GTCAGGATACCTCTCTTGCTCTG	207
*Adamts2*	Forward: GCTCTGCTGAGGCTGTCC Reverse: CATGTGGTATATCGCCGACC	107
*Col1a1*	Forward: GCAACAGTCGCTTCACCTACA Reverse: CAATGTCCAAGGGAGCCACAT	137
*Cxcr6*	Forward: TGGAACAAAGCTACTGGGCT Reverse: AAATCTCCCTCGTAGTGCCC	90
*Mmp13*	Forward: GGTCCTTGGAGTGATCCAGA Reverse: TGATGAAACCTGGACAAGCA	98

### Western blot analysis

Lung homogenates were prepared from C57BL/6 and *Atp8b1* mutant mice (7-9 wks and 14 M ) as described previously [2]. Briefly, 50 μg of total proteins were separated on 10% SDS-PAGE and transferred onto polyvinylidene difluoride membranes. Following blocking in Tris-buffered saline (20 mM Tris·HCl at pH 7.5 and 150 mM NaCl) with 0.1% Tween 20 (TBS-T) containing 5% skimmed milk, the membranes were incubated overnight at 4^◦^C with rabbit polyclonal anti-Nrf2 antibody as per manufacturer's recommendations (Abcam, Cambridge, UK). Following washing of the membrane with TBS-T, membranes were incubated with horseradish peroxidase-conjugated secondary antibody for 30 min at room temperature. This was followed by washes with TBS-T, protein on the membranes was visualized using Pierce ECL Western blotting substrate as per manufacturer's instructions (Thermo Fisher Scientific, Hudson, NH). Horseradish peroxidase-conjugated β-actin antibody (Sigma Aldrich, St Louis, MO) was used as a loading control.

### Statistical analysis

In this study, GraphPad Prism version 10.00 (GraphPad Software, San Diego, CA) was used for statistical analyses. We used Student's T-test to calculate statistical significance between two groups. *p* values < 0.05 were considered to be statistically significant.

### SUPPLEMENTARY MATERIAL TABLES


