# Supplementary material for: Global gene profiling of aging lungs in Atp8b1 mutant mice
Source: Aging (Albany NY). 2016 Sep 29;8(9):2232–50. doi: 10.18632/aging.101056 (PMC5076460; doi:10.18632/aging.101056)
Supplement: Supplementary file 1 [file aging-08-2232-s001.pdf]

SUPPLEMENTARY MATERIAL

Table S1. Aging related overlapping genes in C57BL/6 lungs and *Atp8b1* mutant lungs.

Gene Symbol	Fold change (Atp8b1)	Fold change (C57BL/6)	FDR
2810047C21Rik1	1.006	1.196	0.0512
AKAP13	-1.388	-1.249	0.0398
C130026l21Rik	3.433	2.945	0.0512
CDC73	1.203	1.269	0.0309
COL1A1	-1.924	-1.048	0.0512
COL3A1	-1.213	-1.071	0.0512
CSPRS	2.086	1.761	0.0512
CXCR6	1.742	1.673	0.0512
DDX6	-1.648	-1.819	0.0512
EIF4E3	-1.153	-1.006	0.0512
EPB41L2	-1.438	-1.364	0.0512
EPSTI1	1.268	1.001	0.0512
IGHG3	2.406	1.642	0.0512
IGHG	6.381	4.305	0.0512
IGHM	4.456	1.108	0.0512
LOC664787	1.352	1.676	0.0512
LUZP1	-1.292	-1.228	0.0512
MALAT1	-1.241	-1.062	0.0512
PEG3	-1.772	-1.109	0.0512
PPBP	1.916	1.273	0.0903
PRG2	2.338	1.845	0.0903
PRRC2C	-1.596	-1.184	0.0512
RAPGEF6	-1.178	-1.003	0.0903
RBM5	-1.509	-1.015	0.0512
RIAN	-1.317	-1.405	0.0512
SEMA5A	-1.316	-1.237	0.0512
SF3B1	-1.117	-1.059	0.0512
SLC6A20	1.868	1.117	0.0512
SRRM2	-1.162	-1.014	0.0512
TRA2B	-1.48	-1.122	0.0512

Table S2. Other canonical pathways identified in aged C57BL/6 lungs.

Name	Molecules	-log(p-value)
RhoA Signaling	RAPGEF6,DLC1	7.86E-01
PI3K/AKT Signaling	JAK1,PPP2R2C	7.75E-01
Oncostatin M Signaling	JAK1	7.28E-01
iNOS Signaling	JAK1	6.26E-01
Wnt/β-catenin Signaling	WIF1, PPP2R2C	5.71E-01
EGF Signaling	JAK1	5.35E-01
JAK/Stat Signaling	JAK1	4.44E-01
PDGF Signaling	JAK1	4.21E-01

Table S3. Other canonical pathways identified in aged *Atp8b1* mutant lungs.

Name	Molecules	-log(p-value)
RhoA Signaling	RAPGEF6,PPP1R12A,MYL3,TTN,IGF1,RHOA	1.37E00
Oxidized GTP and dGTP Detoxification	DDX6	1.19E00
Ephrin A Signaling	PIK3CD,PTPN11,RHOA	1.1E00
Clathrin-mediated Endocytosis Signaling	APOC2,PIK3CD,UBD,SERPINA1,IGF1,VEGFC,EPS15	1.09E00
Protein Ubiquitination Pathway	UBE3A,USP36,DNAJC3,HLA-A,USP8,USP2,UBD,B2M,PSMB8	1.07E00
Retinol Biosynthesis	LRAT,LIPC	9.57E-01
Actin Cytoskeleton Signaling	PIK3CD,PPP1R12A,GSN,MYL3,RAC2,TTN, RHOA	7.99E-01
Protein Kinase A Signaling	AKAP13,PPP1R12A,PTPN11,DUSP18,MYL3,PRKCB,CREBBP,PTPN22,NFKBIE,TTN,RHOA	7.95E-01
Superoxide Radicals Degradation	NQO1	7.89E-01
Rac Signaling	PIK3CD,SH3RF1,CYBB,RHOA	7.63E-01
CXCR4 Signaling	RHOH,PIK3CD,MYL3,PRKCB,RHOA	6.48E-01
PDGF Signaling	PIK3CD,STAT1,PRKCB	6.34E-01
Integrin Signaling	RHOH,PIK3CD,PPP1R12A,RAC2,TTN,RHOA	6.29E-01
NF-κB Signaling	TNFRSF17,PIK3CD,PRKCB,CREBBP,NFKBIE	5.62E-01
TNFR1 Signaling	NAIP,NFKBIE	5.6E-01
Calcium Signaling	ATP2B2,SLC8A1,MYL3,CREBBP,ATP2B1	5.3E-01
Death Receptor Signaling	NAIP,TNFSF10,NFKBIE	5.26E-01
Apoptosis Signaling	NAIP,BCL2A1,NFKBIE	5.26E-01
RhoGDI Signaling	RHOH,PPP1R12A,MYL3,CREBBP,RHOA	5.23E-01
Glutathione Redox Reactions	PRDX6	5.03E-01
Unfolded protein response	EDEM1,DNAJC3	5E-01
Ephrin Receptor Signaling	PTPN11,CREBBP,RAC2,VEGFC,RHOA	4.99E-01
mTOR Signaling	RHOH,PIK3CD,PRKCB,VEGFC,RHOA	4.76E-01
Wnt/Ca+ pathway	WNT5A,CREBBP	4.68E-01
EGF Signaling	PIK3CD,STAT1	4.68E-01
Myc Mediated Apoptosis Signaling	PIK3CD,IGF1	4.48E-01
DNA Methylation and Transcriptional Repression Signaling	DNMT3A	4.46E-01
Endoplasmic Reticulum Stress Pathway	DNAJC3	4.46E-01
Signaling by Rho Family GTPases	RHOH,PIK3CD,PPP1R12A,MYL3,CYBB,RHOA	4.39E-01
Xenobiotic Metabolism Signaling	PIK3CD,PRKCB,IL4I1,CREBBP,UGT8,NQO1	3.99E-01
Sonic Hedgehog Signaling	HHIP	3.46E-01
SAPK/JNK Signaling	PIK3CD,RAC2	2.53E-01
TGF-β Signaling	RUNX3,CREBBP	2.53E-01
